# Worldwide assessment of healthcare personnel dealing with lymphoedema

**DOI:** 10.1186/s13561-018-0194-6

**Published:** 2018-04-16

**Authors:** Henrike Schulze, Marisa Nacke, Christoph Gutenbrunner, Catarina Hadamitzky

**Affiliations:** 1 Clinic of Rehabilitation Medicine, Hannover Medical School, Carl-Neuberg-Straße 1, 30625 Hannover, Germany; 2 Cancer Research UK, Beatson Institute, Glasgow, UK; 3 Practice for Lympho-Vascular Diseases, Bahnhofstraße 12, Hannover, Germany

**Keywords:** Lymphology, Lymphoedema, Lymphatic diseases, Medical healthcare resources, Medical education

## Abstract

**Background:**

Lymphoedema is a pandemic with about 250 million people suffering from this condition worldwide. Lymphatic diseases have considerable public health significance, but yet few professionals are specialised in their management causing a substantial burden on health resources.

**Aims and objectives:**

This study aims to give an overview of the approximate number of medical professionals, professional societies, institutions and companies dealing with lymphoedema in various countries. Concepts of improvement for current human resources are considered.

**Methods:**

An online database analysis (Google search engine and PubMed) was carried out for each country of the world. Additionally, relevant congress participant lists as well as member lists of significant medical societies and reports of the World Health Organisation were analysed.

**Results:**

Overall distribution of tertiary level professionals specialised in this field is heterogenous. A decrescent gradient of professionals can be seen between developed and developing countries and between urban and rural areas. Countries in general do not seem to have yet met the current demand for specialists at tertiary level in this field.

**Conclusions:**

This study intends to draw attention to the current medical coverage gaps due to a low number of lymphoedema specialists at tertiary level. It wishes to start a discussion about structured reimbursement and certification of knowledge and skills that are essential incentives for experts to act as multiplicators and change the lack of care in the mid-term. Current fail prescriptions and evitable disability and sick certificates represent a high financial burden that could be reinvested in a correct management. Policy makers must focus in the two above mentioned essential measures. Medical training and the consequent development of the industry will then naturally take place, as it was the case for other professional groups in the past.

## Background

Lymphoedema, also known as elephantiasis, is a chronic disease characterised by massive swelling of limbs or tissues. The disease affects about 250 million patients worldwide [1]. It occurs through accumulation of fluid and other tissue elements that would normally be drained by the lymphatic system. The origin can be genetic or, more commonly, secondary to injuries.

Secondary causes of lymphoedema vary according to geographic distribution [2]. In developing countries, it is often a corollary of parasitic infections through Filaria or Loa Loa or caused by Podoconiosis and has massive global public health significance [3].

In developed countries, secondary lymphoedema mainly occurs after cancer therapy [4]. It is strongly associated with prevalent cancer types, e.g. breast or prostate cancers [5]. Depending on the type and extent of treatment, the anatomic location, the heterogeneity of assessment methods, and the follow-up, the reported incidence of cancer-related lymphoedema varies [6, 7]. Because lymphatic swelling can occur decades after cancer treatment, cancer patients undergoing lymph node dissection should be considered at lifetime risk.

Despite its epidemiological relevance, only few professionals are specialised in the management of lymphatic diseases [8–10]. Lymphology is a mostly overlooked field in medical schools and exposure of medical professionals to this field varies greatly [10, 11]. The lack of professional knowledge especially at the level of general practitioners, as first medical contacts, is considered to be a problem that impacts early detection, leads to misdiagnosis and inappropriate treatment and results in poor management of health resources [9, 10, 12–17]. Natural exacerbation of the swelling causes increased demand on healthcare resources but can also lead to life threatening infections as well as psychosocial exclusion followed by a diminished quality of life or even depression [18–21].

Appropriate management of lymphoedema, including surgical procedures, can cure the condition in 30–40% of the cases [22] but also conservative therapy with manual lymphatic drainage and compression leads to an improvement of patients’ quality of life and increases their presence at work [23, 24]. Although the number of healthcare professionals specialised in lympho-vascular diseases seems to be insufficient, this phaenomenon has been left unquantified until now. In the literature of the last decade only a single publication [13] directly addresses the discrepancy between offered and demanded services in this field of healthcare. Nevertheless, medical professionals could improve the current healthcare situation through their multiplication characteristics by influencing the interdisciplinary work with nurses and/or physiotherapists regarding the chosen therapy and by spreading rare knowledge in universities, hence promoting research. Empowerment of future generations could create a stronger relationship between the local health services and lymphoedema patients in the future [25].

The objective of this study was to gain an overview of the approximate number of medical physicians and researchers currently dealing with lymphoedema. Another aim was to assess the ratio between healthcare professionals and general population in vast geographical areas, and this despite low numbers of specialised healthcare professionals with tertiary level of training to be found in this field of rare knowledge. Additionally, professional societies and companies associated with lymphoedema were counted in order to understand possible concepts of improvement for current human resources, as professional training and certification allow further development in this important field. Furthermore, potential policy implications such as structured reimbursement or higher public research funding were included in the discussion of this study.

## Methods

In order to map medical professionals, societies, institutions and industrial companies dedicated to lymphoedema, research was performed exclusively online for each single country of the world in three independent phases. The targeted professional groups were physicians and researchers. The search included published data from 2005 to 2015 and was restricted to six languages, namely English, German, French, Spanish, Portuguese and Italian.

**Fig. 1:**
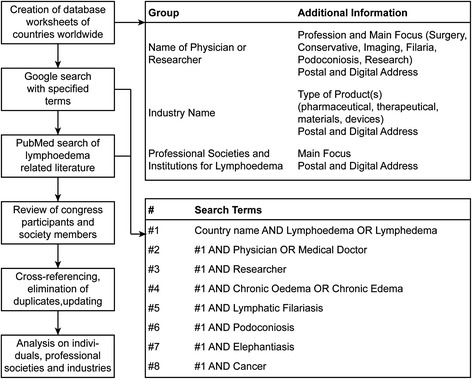
Flowchart showing the systematic data search process including information on database structure and search terms

In the first phase, a data collection was performed with the Google search engine. For each single *country name*, the word lympho*edema was introduced, and the top ten listed websites were analysed. Then, these terms were successively combined with the following words: *physician* or *medical doctor*, *researcher*, chronic o*edema, *lymphatic filariasis*, *podoconiosis*, *elephantiasis* and *cancer *(Fig. 1). It also allowed retrieving information on professional societies and industrial companies.

In the second phase, the search was conducted with the same keywords in the PubMed database. Relevant studies from the database were identified by their titles and abstracts. In case of uncertainties, the full text was retrieved and authors and participating institutions were listed in the corresponding countries.

In the third phase, duplicates were removed. Cross analysis on congress participants listed in scientific meetings dealing with the topic of lymphatic diseases was performed and relevant reports from the WHO were analysed. If available, member lists of medical societies for lymphatic diseases were included in the analysis. The quantifications of specialised professionals were not performed on numeric data, but on the personal data available online for each expert, including affiliation, address, and fields of interest.

### Data extraction and analysis

A data extraction form was developed in Excel. Individual physicians and researchers were assigned to one of the following categories depending on their main focus: *surgery*, *conservative therapy*, *research*, *filariasis*, *podoconiosis* and *medical imaging.* Their professional affiliations were recorded along with the postal and digital address. When the information was originated in research papers, experts were grouped by geographic distribution, not by paper. International research groups had their members therefore grouped separately according to their country of residence. Experts were counterchecked individually for their qualifications (medical doctors or e.g. biologists) and a Google search for data under their name was performed to assign them to a main focus as stated above. Medical societies and institutions were also sorted by country as well as main focus and likewise pharmaceutical companies (Fig. 1). The main findings were analysed narratively as statistical pooling was not adequate for this scoping study.

Ethics approval was not required for this study, as online information is publicly accessible and there is no reasonable expectation of privacy.

## Results

The conducted research included 208 countries on six continents. Of the entirety of countries in the world, 123 countries provided online information on at least one of the researched topics (59% of the total sample). Activities could be mapped in twelve out of 23 countries in North and Central America; 13 out of 17 in South America; 27 out of 49 countries in Europe; 14 out of 20 countries in Oceania; 25 out of 48 Asian countries and 32 out of 51 countries in Africa.

**Fig. 2:**
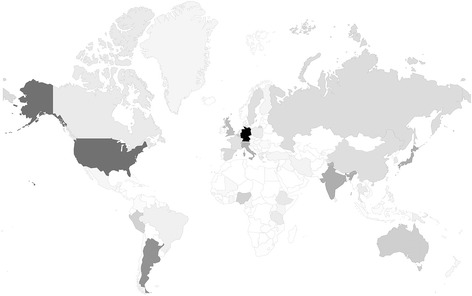
Absolute distribution of professionals exclusively at tertiary level dedicated to lymphatic diseases across the world. The colour code scale shows white for zero professionals (e.g. Afghanistan or Kazakhstan) and black corresponds to the maximum number of 432 professionals found in Germany. The higher the absolute number of tertiary level professionals in a country, the darker the colour

**Fig. 3:**
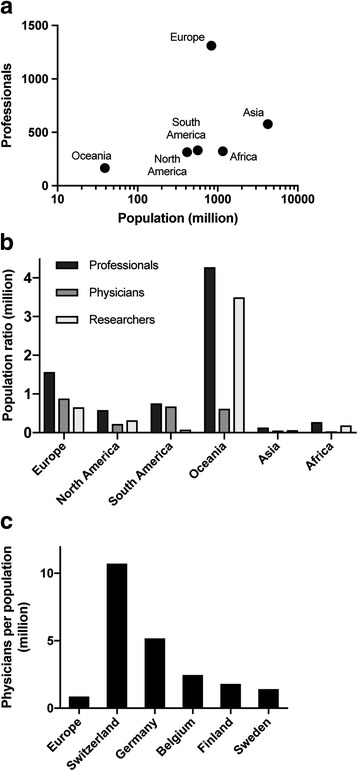
**a** Scatter plot of logarithmic ratio between tertiary level professionals and population for each continent, **b** Ratio of tertiary level professionals, physicians and researchers per million inhabitants for each continent, **c** Countries in Europe presenting the highest ratio of physicians per million inhabitants (bar chart)

First, the exact numbers of tertiary level professionals dedicated to lymphatic diseases were analysed (Fig. 2). As expected, their distribution is very heterogeneous, e.g. Pakistan, with a population of more than double than Germany, has only four listed professionals, the latter having 432. A high absolute number of professionals could be tracked in Europe (*n* = 1310) (Fig. 3a*;* Table 1). Whilst being the continent with the lowest absolute number of tertiary level professionals currently working in the field of lymphology, Oceania has the highest ratio of professionals per capita (165 professionals per 38.5 million people) (Fig. 3a and b*,* Table 1). Europe, on the contrary, displays only an average level of coverage of its population of half a billion notwithstanding the high absolute numbers of professionals. Asia, on the lower extreme, has a coverage ratio of tertiary level professionals per million inhabitants below one (Fig. 3b*,* Table 1).

**Table 1:** Data for each continent for Fig. 2

Continent	Professionals^a^	Physicians	Researchers	Population (in million)	Professionals/population (per million)	Physicians/population (per million)	Researchers/population (per million)
Europe	1310	739	550	833.9	1.57	0.89	0.66
North America	332	128	182	562.6	0.59	0.23	0.32
South America	314	281	33	414.1	0.76	0.68	0.08
Oceania	165	24	135	38.5	4.28	0.62	3.50
Asia	577	253	298	4222.9	0.14	0.06	0.07
Africa	323	26	221	1159.8	0.27	0.02	0.19

^a^Professionals are regarded as physicians, researchers and also other persons involved in the topic of lymphoedema such as stakeholders at the World Bank who provide money for lymphoedema research and education

Additionally, we split the focus of the tertiary level professionals into clinical activity and research (Fig. 3b*;* Table 1). Professionals with both foci were included in both statistics. When considering only clinically active physicians per million inhabitants, e.g. Europe had high ratios (0.89 ppm), whereas Africa, with a total of 26 clinically active physicians, has according to our research criteria practically inexistent medical coverage in this field. Similar coverage exists for researchers in most continents excluding Oceania. Oceania has a high ratio of researchers per million inhabitants (3.5 ppm).

**Fig. 4:**
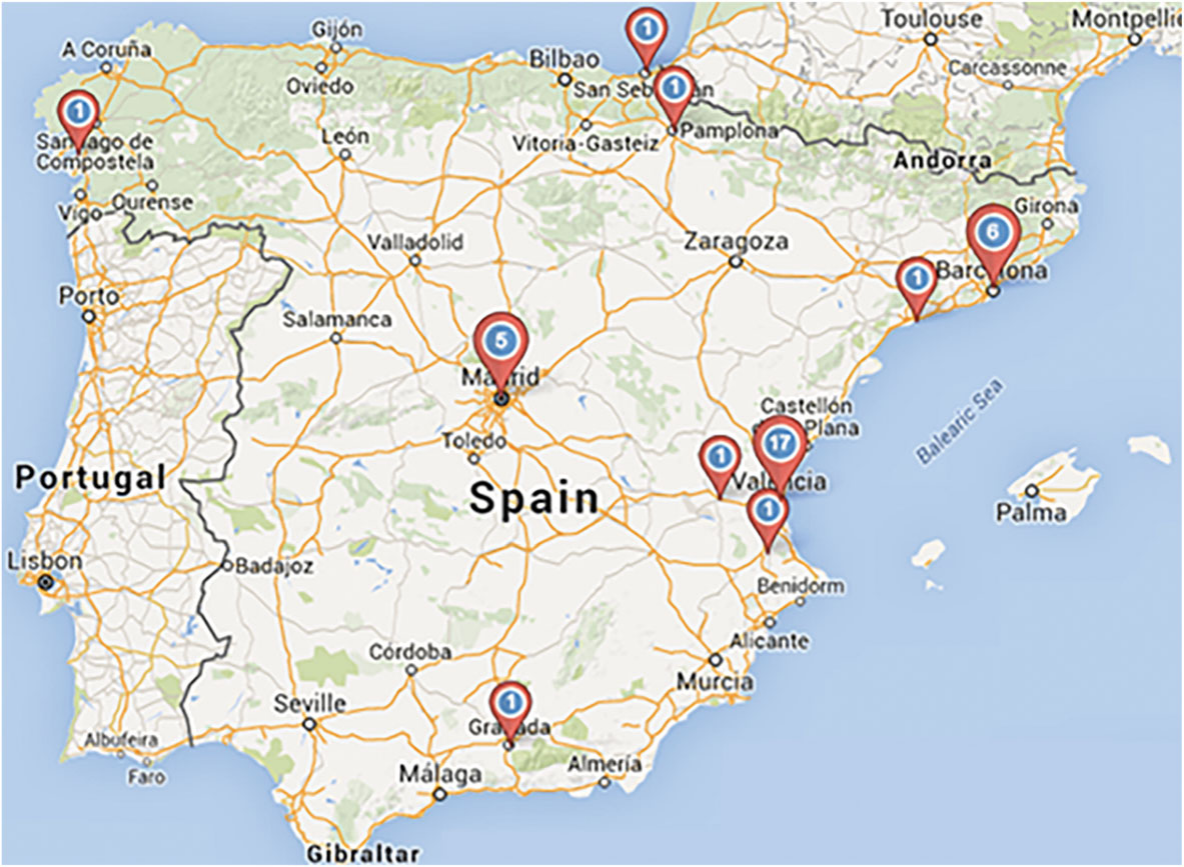
Geographical distribution of physicians in Spain in absolute numbers

Also, in Europe inequality is such, that some countries presented very poor medical coverage of lymphoedema despite belonging to the European continent. Differences can already be seen comparing five European countries (Fig. 3c). Interestingly, countries with a large population such as Japan do not seem to reach the higher ratios of less populated countries like Switzerland (Table 2). The uneven distribution of tertiary level physicians was not only present at a global level, but also within single countries. Spain, with a total of 34 active physicians, can be considered a paradigm of depletion in rural areas with most physicians to be found in Madrid (*n* = 5), Valencia (*n* = 17) or Barcelona (*n* = 6) (Fig. 4).

**Table 2:** Overview of the leading countries and ratios per continent of professionals, physicians, researchers and societies

	Professionals	Physicians	Researchers	Professionals/population (per million)	Physicians/population (per million)	Researchers/population (per million)	Societies/Population (per million)
Europe	Germany	Germany	UK	Switzerland	Switzerland	Belgium	Switzerland
Highest quantity / ratio	432	421	79	13.80	10.74	5.70	6.22
Population (in million)	80.9	80.9	64.5	8.2	8.2	11.2	8.2
North America	USA	USA	USA	Grenada	Grenada	Grenada	Grenada
Highest quantity / ratio	241	75	148	47.01	18.81	28.21	9.40
Population (in million)	318.9	318.9	318.9	0.1	0.1	0.1	0.1
South America	Argentina	Argentina	Brazil	Argentina	Argentina	Guiana	Bermuda
Highest quantity / ratio	181	168	15	4.21	3.91	2.62	15.34
Population (in million)	43.0	43.0	206.0	43.0	42.0	0.7	0.1
Oceania	Australia	Australia	Australia	Amer. Samoa	Amer. Samoa	Amer. Samoa	Tuvalu
Highest quantity / ratio	80	10	70	162.36	18.04	144.32	101.08
Population (in million)	23.5	23.5	23.5	0.1	0.1	0.1	< 0.1
Asia	Japan	India	Japan	Micronesia	Japan	Micronesia	Micronesia
Highest quantity / ratio	138	102	78	9.61	0.47	9.61	9.61
Population (in million)	127.1	1295.3	127.1	0.1	127.1	0.1	0.1
Africa	Nigeria	Cameroon	Nigeria	Sao Tome	Comoros	Sao Tome	Sao Tome
Highest quantity / ratio	76	3	72	21.47	2.60	21.47	10.73
Population (in million)	177.5	22.8	177.5	0.2	0.8	0.2	0.2

Data regarding the specialty of physicians (Table 3) showed that most are focused on the conservative management of the disease (*n* = 641), most of them being located in Europe (*n* = 535). Concerning the operative therapy of lymphoedema, our online search was able to identify the highest number of surgeons in Europe (*n* = 295), followed by Asia (*n* = 139). Surprisingly, very few physicians are specialised in imaging procedures of lymphatic vessels (*n* = 64) despite the overlap with imaging methods used in cancer management. Concerning filarial lymphoedema, only 40 specialists could be mapped. Our search led to just two medical specialists dedicated to podoconiosis. In Asia (*n* = 163) and Oceania (*n* = 21) most physicians additionally are researchers, showing a strong potential for innovation in these continents. This is also true for North America.

**Table 3:** Distribution of physicians’ specialties in the field of lymphology according to continent

	Surgery	Conservative Therapy	Medical Imaging	Filaria	Podoconiosis	Research
Africa	8	3	0	4	0	**16**
Asia	139	28	27	21	0	**163**
Oceania	6	5	0	5	0	**21**
Europe	295	**535**	25	5	1	234
North America	**68**	36	10	4	0	**68**
South America	**61**	34	2	1	1	5
**Total**	577	641	64	40	2	507

The highest numbers of physicians’ specialists of each continent are marked bold

Our search regarding professional societies and institutions dedicated to this field of medicine showed that the leading single country is the U.S. with 161 societies (Table 4). European countries altogether have 598 societies mentioning lymphoedema as one of their points of interest, all specialties included, but oftentimes the professionals involved in these societies or institutions are the same at regional, national and international level. Additionally, some Asian countries as India are well organised in this field with a total online track of 96 Indian societies. Table 4 also shows where industrial companies dedicated to lymphoedema products are located. Leading countries are the U.K. (*n* = 33) and the U.S. (*n* = 42). Respectively, most companies can be found in Europe (*n* = 90) and North America (*n* = 49). Developing countries are virtually depleted of industrial companies in this field, forcing them to import needed products or simply failing to provide them.

**Table 4:** Overview of societies, institutions and industry specialised in lymphatic diseases per continent including leading countries

	Europe	North America	South Amercia	Africa	Asia	Oceania
Professional societies and institutions	598	218	54	138	319	75
* Leading country*	*Germany: 88*	*USA: 161*	*Argentina: 33*	*Ethiopia: 16*	*India: 96*	*Australia: 31*
Industry	90	49	none	3	6	2
* Leading country*	*UK: 33*	*USA: 42*	*none*	*Kenya, Tanzania, South Africa: 1*	*Taiwan: 3*	*Australia: 2*

## Discussion

The social burden of the lymphoedema pandemic is inversely proportional to the low number of medical professionals dedicated to its management or research. It is translated in inadequate prescriptions, poor management of health resources, as well as time of work absenteeism, social exclusion and stigma of patients [12, 18, 19, 25–27]. The conducted study aimed at a semi-quantification of the problem by showing where medical professionals can be found, and where their coverage must be expanded.

### Aspects of online search methods

One possible limitation of our study resulted from restricting our search to the internet. Certainly, local offers of lymphoedema management exist that were not visible online. About 67% of the world’s population does not have access to the internet [28]. Especially in developing regions patients still seek advice from traditional healers [29–35]. This is supported by reports from the Caribbean, showing that most patients only seek care from university-trained but more expensive healthcare providers when traditional treatment has shown to be ineffective [26, 27]. If patients and their communities are taught about their disease, it has been shown that a loco-cultural understanding of the disease can coexist with the biomedical one [36]. Therefore, additional low-cost programs should be established, as they improve lymphoedema outcomes, even leading to an important economic benefit as patients are able to work again or work more [24]. Examples of low-cost interventions are training of communities on hygiene measures of the lymphoedema limb as well as demonstrations for the application of topic antibiotics on secondary wound infections and bandaging. When comparing the yearly costs per patient in hygiene and/or bandaging materials including antibiotic ointments (direct healthcare costs) with the gain in working days/salary since program implementation (indirect workforce costs), Stillwaggon et al. [24] could calculate per-person savings of more than 130 times the per-person costs of their program. This demonstrates the importance of understanding the cultural background when planning health resources in these societies [37]. The online selection bias might nevertheless have the advantage of listing only professionals that have reached a certain degree of impact, at least at regional level. This study therefore mimics the barriers encountered by lymphoedema patients seeking healthcare providers with the support of online search engines but might not be exact concerning the current local healthcare measures provided by the communities.

### Language bias

Considering the language barrier, the analysis was limited to six languages spoken by the main authors. Regions of the world not using the latin alphabet were impermeable to our search thus creating false negatives mainly in the data on Asia and North Africa. Therefore, although our search was thoroughly performed with the name of every single country in the world, the fact that we were using western languages in our search reduced our scope of investigation extensively, especially considering that Asia is an extremely populated region of the world. Retrospectively, most countries failing to provide any online information were small in size and population. Additionally, some of these countries use specific local terms to name lymphoedema. Common synonyms are “big foot” in Haiti [29] or “mossy foot” in Ethiopia [38] (Table 5). These alternative terms might have led to incomplete listing of involved professionals in the respective regions of Africa, Central America and Asia.

**Table 5:** Overview of local dialect terms and their medical translation for symptoms of lymphatic diseases

Local term	General term	Country
*Gwo pye* (Creol dialect) or *big foot*	Elephantiasis	Haiti [29]
*Maklouklou* or *gwo gren*	Hydrocele	Haiti [29]
*Dicipela*	Erysipela	Dominican Republic [26, 32]
*Seca*	Large boil in the groin or upper thigh	Dominican Republic [26, 32]
*Mossy foot*	Podoconiosis	Ethiopia [38]
*Biaye or edoa*	Inguinal lymphadenitis	Ghana [30]
*Ahu par* or *esam*	Fever with chills and rigors accompanied by swollen groin glands and legs	Ghana [30]
*Gyepim, dubah* (both in Ahanta and Nzema dialect) or *large leg*	Elephantiasis	Ghana [30]
*Etow (Ahanta), wheba* (Ahanta), *tuekeh* (Nzema) or *edoma holokpoo*	Hydrocele	Ghana [30]
*Yanaikkal*	Lymphoedema or elephantiasis of the lower leg	India [56]
*Veraveekkam or veravadam*	Hydrocele	India [56]

### Importance of developing adequate training offers

General analysis of the absolute number of professionals qualified at tertiary level in this field reveals a great need for Asian, North American and European countries to organise training offers. In the reminiscent regions, countries do not seem to possess enough professionals to offer the whole spectrum of medical training on lymphatic diseases (Fig. 2*;* Table 3). Therefore, it is paramount that healthcare providers in Asia, North America and Europe embrace this challenge, take responsibility and promote training offers to improve global geographical coverage. Lymphology should already be taught in undergraduate medical school in order to close the knowledge gap [11]. Analysing general medical care in most countries of Africa shows that although this continent is responsible for 24% of the burden of diseases worldwide, it is only disserved by 3% of the total healthcare providers [39]. One could postulate that if financial resources invested in humanitarian treatment of disease in developing countries in the past had instead been used for medical training, the present situation might not be as unbalanced. In these countries, in spite of an established prevalence of 40 million patients affected with lymphoedema as a consequence of lymphatic filariasis, few are able to receive proper medical support and treatment [9, 10, 40], especially as the patients are more likely to get the disease if they have a low or medium socioeconomic status and might therefore not be able to afford treatments [41, 42]. On the other hand, the treatment of filariasis constitutes one of the success stories of mass drug administration (Global Programme to Eliminate Lymphatic Filariasis - GPELF) with decreased primary infections following the global programs of eradication [43]. Community healthcare workers carried out the pharmacologically driven programs. Nevertheless, healthcare workers were not specifically informed about lymphoedema and lacked the clinical expertise of tertiary level professionals to diagnose this condition as a possible corollary of filaria infection. This constituted a lost opportunity of broad geographic impact perpetuating inadequate management, poor treatment and failure to meet the specific needs of patients with lymphoedema after filarial infection [26, 27, 31, 44, 45]. But, even so, substantial health and economic benefits have risen from the program, especially in what concerns lymphoedema prevention by treating the causal worm [42]. National strategies are often followed by new investments. The government of Ethiopia just recently invested in the fight against neglected tropical diseases (including lymphoedema/podoconiosis) in order to achieve the goal of eliminating these until 2020 according to a set up national plan [45]. An important addition to national strategies would be to collect statistical data on the current prevalence of the disease and apply epidemiologic information in the creation of medical support where help is needed [46, 47].

The heterogeneity of medical training is not restricted to developing countries. A lack of specialised physicians especially exists in poorer, more rural and remote areas as described for Australia [48] and similar findings were postulated even across the U.K. [8]. In the case of Spain, most mapped physicians were located in cities. Lymphoedema consultation services are mostly based in hospitals and for public insurance patients the access is restricted, making it more difficult to seek qualified treatment [8]. With the exception of some regions of Germany and Switzerland, professionals specialised in lymphatic diseases were very seldom general practitioners, rendering this distributary problem a characteristic of most countries worldwide.

### Lymphoedema researchers

Regarding the data on the number of researchers dedicated to lymphoedema, one of our main difficulties during our search was to identify if these professionals were physicians, or belonged to other professional branches e.g. biologists. Nevertheless, this academic distinction has little influence on the capacity of researching solutions for a disease, as this plurality might be beneficiary for innovation. Most researchers originate from continents with developed countries such as Oceania or Europe. Awareness and public funding of research are still low on upcoming continents such as South Asia although things have started to change [49]. Young researchers are less attracted to investigate a field they have never heard about as most of their universities have failed to include lymphatic diseases in the university curricula. An important milestone of a global strategy to address the problem of medical care of lympho-vascular diseases should also include measures to break the vicious circle of the lack of research. This has to be achieved outside of the laboratories at sub graduate level through innovative training offers.

### Professional societies including lymphatic diseases in their topics of interest

Patients trying to find qualified medical healthcare professionals online, will be confronted with similar limitations encountered in this study. Nevertheless, patients express a deep need for guidance and information [50]. A medical doctor with expertise in lymphatic diseases that cannot be found by patients searching online is equivalent to a very localised resource. Therefore, we also analysed the number of professional societies, as they play a paramount role in providing information on the spectrum of expertise of their members. Physicians were easily found in South America as the Pan-American Society for Lymphology and Phlebology published a list of physicians. Other examples of societies providing well-structured information are to be found in Germany and Switzerland. There was no clear correlation between the number of professionals specialised in lymphology in a region or country, and the number of professional societies active in this field. But most problematic was the complete lack of independent societies for lymphatic diseases in most countries of the world that could contribute to high level postgraduate education and facilitate discussions about diagnosis and treatment guidelines.

### Industrial activities in lymphology

In our analysis of the distribution of industrial activity connected to lymphoedema, North America and Europe were the leading continents. The associated industry is currently not pharmaceutical but mainly biotechnological focussing on measurement devices, surgery microscopes and compression garments. Similarly to pharmaceutical companies, biotechnology companies show less interest in e.g. Africa or South America most likely due to poor distribution networks and low profit margins.

### Additional policies for palliation of current deficits

It is somewhat striking to see this disparity of numbers of specialised physicians in developed countries. We believe the reasons for their relatively high number in some countries as e.g. Belgium or Germany to be historical. The first lymphoedema clinic was founded in Germany and already in the late seventies, German healthcare insurances started reimbursing lymphoedema therapy modalities. Although many physiotherapists in Germany are trained in manual lymphatic drainage, only medical doctors are entitled to prescribe it. Therefore, there is a positive peer pressure between both professional groups. This leads to some degree of medical training in this field [51], mostly of general physicians as well as doctors in physical and rehabilitation medicine [52]. Why do other developed countries with a similar number of lymphedema patients still lack specialised human health resources? Improving the quality of management of this disease in Germany and the Benelux was not solely due to medical professionals in itself but mostly to the professional group of the physiotherapists. Here, reimbursement of lymphoedema treatment was adequate, but only allowed to those physiotherapists having undergone validated specialised training. This motivating restriction never happened in the medical profession. The medical chamber of doctors in Germany failed until now to recognise a medical subspecialty in lymphatic diseases, although reiterated proposals have been delivered by professional societies since the nineties. Until today, all physicians are entitled to manage lymphoedema patients. Also, the reimbursement for this medical act is very low and calculated for periods of three months regardless of the number of times the patient has to be seen in that time interval, leading to low motivation to treat this chronic disease. Paradoxically, European policy makers do not seem to be aware of the costs of fail prescriptions and poor management.

In the U.S. severe reimbursement barriers still exist for lymphatic diseases [18, 53]. Here, not only direct costs, but also travel costs and loss of earning prohibit patients to seek proper medical treatment [25, 54].

Interestingly, in the U.K., where the number of medical doctors specialised in this field is also below demand, nurses established a culture of lymphoedema treatment based on patient self-management and training of family members to provide lymphatic drainage. Showing patients how to manage their lymphoedema reduces exacerbations and improves their quality of life [24, 55]. Therefore, it is not neutral to decide which professionals will become key players in this field. It is not sufficient to limit the reimbursement to certified professionals, but special incentives for patient training and empowerment should also be promoted. They eventually increase the patients’ independence from the same providers of care that are so scarce in most health systems.

## Conclusions

Lymphoedema patients oftentimes experience a chaotic journey from diagnosis to treatment due to scarce support by the medical system. The time prior to diagnosis and thereafter prior to health reimbursement must be shortened. Interventions in this health sector would ideally be based on the knowledge of the extent of the problem. The present study represents a first attempt to quantify a group of professionals characterised by a deficiency of data. Not only the medical expertise in lymphology is not officially recognised as a subspecialty (except for the Netherlands and the United Kingdom) and experts are therefore not listed as such in the corresponding professional chambers, but also the number of professionals specifically offering health services in this field is globally very limited. With the right policies, as certification and homologation of knowledge at tertiary level, restriction of patient care solely to specialised healthcare providers, structured reimbursement and sufficient research funding, the current situation can be improved. Targeted incentives for medical professionals trained in this field would then ameliorate the management of these diseases, optimise the use of already available financial resources, promote the passage of knowledge and skills in universities, promote research, empower patients and influence interdisciplinary work. These professionals can potentially become the true motors of a worldwide solution. The authors of the study strongly support the use of their database for further research according to the principles of share of good practice.

## Abbreviations

ppm: Parts per Million
WHO: World Health Organization
